# Dr. Electa B. Whipple

**Published:** 1907-11

**Authors:** 


THE Physicians' League recently took action on the death of Dr. Electa B. Whipple in adopting the following:

WHEREAS, It has pleased our Heavenly Father in his infinite wisdom to call to eternal rest our friend and co-laborer, Dr. Electa B. Whipple, a woman whose noble Christian character and high standard of professional life were an inspiration to her associates; and

WHEREAS, The Physicians' League, of which organisation she was the first president, feels deeply the loss it has sustained, therefore be it

*Resolved*, That the League continue its annual contribution to the College Creche as a tribute to her memory, and that a copy of these resolutions be sent to her friends; to the Buffalo Medical Journal; and be spread on the minutes of this society, the Physicians' League.

JANE W. CARROLL,
MARY INNIS DENTON,
M. ELIZABETH SCHUGENS.
Committee.

